# Mechanisms of Matrix-Induced Chemoresistance of Breast Cancer Cells—Deciphering Novel Potential Targets for a Cell Sensitization

**DOI:** 10.3390/cancers10120495

**Published:** 2018-12-06

**Authors:** Bastian Jakubzig, Fabian Baltes, Svenja Henze, Martin Schlesinger, Gerd Bendas

**Affiliations:** Pharmaceutical Institute, University of Bonn, An der Immenburg 4, 53123 Bonn, Germany; bastian.jakubzig@uni-bonn.de (B.J.); fabian-baltes@uni-bonn.de (F.B.); svenja.henze@uni-bonn.de (S.H.); martin.schlesinger@uni-bonn.de (M.S.)

**Keywords:** breast cancer, CAM-DR, CREB, cisplatin, collagen, FAK, integrin, MAPK, mitoxantrone, Wnt signaling

## Abstract

Tumor cell binding to microenvironment components such as collagen type 1 (COL1) attenuates the sensitivity to cytotoxic drugs like cisplatin (CDDP) or mitoxantrone (MX), referred to as cell adhesion mediated drug resistance (CAM-DR). CAM-DR is considered as the onset for resistance mutations, but underlying mechanisms remain elusive. To evaluate CAM-DR as target for sensitization strategies, we analyzed signaling pathways in human estrogen-positive MCF-7 and triple-negative MDA-MB-231 breast cancer cells by western blot, proteome profiler array and TOP-flash assay in presence of COL1. β1-Integrins, known to bind COL1, appear as key for mediating COL1-related resistance in both cell lines that primarily follows FAK/PI3K/AKT pathway in MCF-7, and MAPK pathway in MDA-MB-231 cells. Notably, pCREB is highly elevated in both cell lines. Consequently, blocking these pathways sensitizes the cells evidently to CDDP and MX treatment. Wnt signaling is not relevant in this context. A β1-integrin knockdown of MCF-7 cells (MCF-7-β1-kd) reveals a signaling shift from FAK/PI3K/AKT to MAPK pathway, thus CREB emerges as a promising primary target for sensitization in MDA-MB-231, and secondary target in MCF-7 cells. Concluding, we provide evidence for importance of CAM-DR in breast cancer cells and identify intracellular signaling pathways as targets to sensitize cells for cytotoxicity treatment regimes.

## 1. Introduction

The loss in sensitivity of tumor cells against antineoplastic treatment regimes, referred to as chemoresistance, remains the major obstacle in the clinical treatment of cancer patients [1]. In the majority of cases, resistance is based on a genetic reprogramming of tumor cells resulting in mutated signaling pathways to prevent apoptosis or in a modified trafficking or intracellular processing of cytotoxic drugs, often associated with enhanced drug efflux mechanisms [2].

However, the embedding into a protective microenvironment appears as an initial process and premise for subsequent mutations of tumor cells. This first step, based on tumor cell binding to components of the extracellular matrix (ECM) such as collagen, laminin, or fibronectin, is regarded as rapid process to induce an early adaptation for prolonged tumor cell survival. Thus, cell adhesion mediated drug resistance (CAM-DR) appears as functional onset to escape cytotoxic stress for different tumor cell entities. Although CAM-DR has initially been described in multiple myeloma and other malignancies of hematopoietic origin [3], which has been related to the minimal residual disease phenomenon [4], some recent reports refer to CAM-DR in solid tumors, too. This seems highly relevant for those solid tumor entities, which are rich in matrix tissues, such as ovarian cancer [5], or oral squamous cell carcinomas [6].

Considering the extensive collagenous areas of e.g., collagen type 1 (COL1) surrounding the lobules forming the connective tissue in the mammary gland, breast cancer appears highly probable to undergo CAM-DR and to make use of this mechanism. Although breast cancer patients initially respond well to guideline-based cytotoxic drugs, such as mitoxantrone (MX), doxorubicin, or platinum drugs, a significant percentage of patients sustain the disease and relapse within a five-year period. Recurrence of breast cancer often goes along with an attenuated sensitivity towards these drugs [7]. Breast cancer mainly differs in its surface receptor profile, i.e., the appearance of estrogen, progesterone receptors or HER2, which can be therapeutically targeted. Notably, various breast cancer cell lines are also known to be differently influenced by COL1. Whereas COL1 promotes adhesion and spreading in estrogen receptor positive MCF-7 and triple negative MDA-MB-231 cells, MCF-7 cells become stiffer, while MDA-MB-231 cells grow more elastically [8].

However, the molecular mechanisms of CAM-DR and underlying signaling pathways appear elusive. Considering the key role of CAM-DR for enabling a genetic resistance formation, an insight into the molecular basis of CAM-DR could offer attractive novel targets for an early interference with resistance formation by pharmacological sensitization strategies.

Integrins, a family of heterodimeric adhesion molecules with ubiquitous cellular expression, represent the most important group of candidates to be involved in CAM-DR. Upon binding to the ECM, integrins regulate not only physical cell attachment, but also activate specific signaling pathways, which can enhance tumor cell invasion, proliferation and survival [9]. Although those protumorigenic effects of integrins in breast cancer have not been considered in terms of CAM-DR, a link between integrins and malignancy of breast cancers could be elucidated. Amongst others, a doxorubicin and epirubicin-resistance in MDA-MB-231 and Hs578T breast cancer cells was found to be related to integrin signaling [10,11]. In these terms, α_ʋ_-integrins were shown to induce taxol resistance in MCF-7 cells by affecting the cell cycle regulation [12]. Nevertheless, the mechanisms by which integrins affect the chemosensitivity appear to be highly diverse and far from being generalizable. Consequently, an interference with the integrin signaling axes appears promising for sensitization strategies, since resistance formation could thus be disturbed before genetic modifications occur. Furthermore, integrin signaling pathways should offer multiple pharmacological targets and flexibility for adaption.

Considering integrin signaling pathways, the phosphatidylinositol 3-kinase (PI3K)/protein kinase B (AKT) pathway turned out to be of general importance for malignancy of tumors [13,14,15]. The PI3K/AKT pathway can be activated upon integrin binding causing activation of focal adhesion kinase (FAK) and in turn the recruitment of Src protein kinases [13,14,15,16], which has been associated with increased survival of e.g., ovarian cancer cells [17,18]. PI3K and its downstream effector AKT thus impact on various cellular pathways affecting cell growth, proliferation and survival.

One of these pathways is Wnt signaling via phosphorylation of the glycogen synthase kinase 3β (Gsk-3β). Wnt signaling deregulation is often associated with tumorigenesis [19]. In a recent study, we reported that integrin activation in melanoma cells associated with loss in sensitivity to cisplatin (CDDP) leads to upregulated Wnt signaling [20].

Integrin binding is also associated with FAK dependent activation of the mitogen-activated protein kinase (MAPK) pathway [21], which is considered a key regulator of cell proliferation and growth [22]. Interference with this pathway has been shown to alleviate CAM-DR phenomena in hematopoietic cells [23].

MCF-7 and MDA-MB-231 breast cancer cells display a loss in sensitivity against mitoxantrone (MX) and cisplatin when cultivated on a COL1 matrix. Applying proteome profiler arrays, mapping the status of human kinases in both cell lines treated with MX and COL1, we investigated different β1-integrin related signaling molecules to explain underlying mechanisms of CAM-DR. Thereby, we elucidated potential targets for sensitization strategies and were able to show that blocking FAK or PI3K is efficient to sensitize MCF-7 cells to MX and CDDP. Data indicate that Wnt signaling is not involved in the indicated experimental resistance formation. Based on a β1-integrin knockdown approach in the MCF-7 cell line, we detected a shift to the MAPK pathway, which is even more active in EGFR expressing MDA-MB-231 cells [24]. Consequently, blocking Extracellular-signal Regulated Kinase (ERK) and cAMP response element-binding protein (CREB), the latter being highly upregulated in both cell lines upon COL1 binding, appears to be a promising approach. Our findings provide evidence for the importance of different CAM-DR mechanisms depending on the respective breast cancer cells and reveal potential targets originating from the integrin signaling routes.

## 2. Results

### 2.1. Deregulation of Signaling Pathways in MCF-7 and MDA-MB-231 Cells Upon Cultivation on COL1 and MX Treatment

Human MCF-7 and MDA-MB-231 breast cancer cells display a higher resistance against CDDP and MX cytotoxicity, when cultivated on a COL1 coated surface, compared to cultivation on uncoated surfaces. This becomes evident by a shift in the MCF-7 cells’ EC_50_ values from 18.0 µM (pEC_50_ = 4.74 ± 0.27) for plain cells to 21.3 µM (pEC_50_ = 4.67 ± 0.15) for COL1 treated cells in case of CDDP, which is in case of MDA-MB-231 a shift from 28.7 µM (pEC_50_ = 4.54 ± 0.21) to 37.3 µM (pEC_50_ = 4.43 ± 0.14). MCF-7 cells show a change from 0.115 µM (pEC_50_ = 6.94 ± 0.32) to 0.147 µM (pEC_50_ = 6.83 ± 0.27) for MX, while MDA-MB-231 move from 0.203 µM (pEC_50_ = 6.69 ± 0.14) to 0.284 µM (pEC_50_ = 6.55 ± 0.13). These findings indicate an adhesion based mechanism, most probably as result of an integrin mediated process. All known COL1 binding integrins contain a β1 subunit recognizing the GFOGER sequence of COL1 [25], which consequently has to be kept in mind when considering COL1 mediated effects. Because of the roughly 100 times lower concentrations of MX compared to CDDP to induce cytotoxicity in both cell lines, we put our main focus on MX based strategies in MCF-7 cells and complemented these by CDDP data, whenever cells showed promising responses.

**Figure 1:**
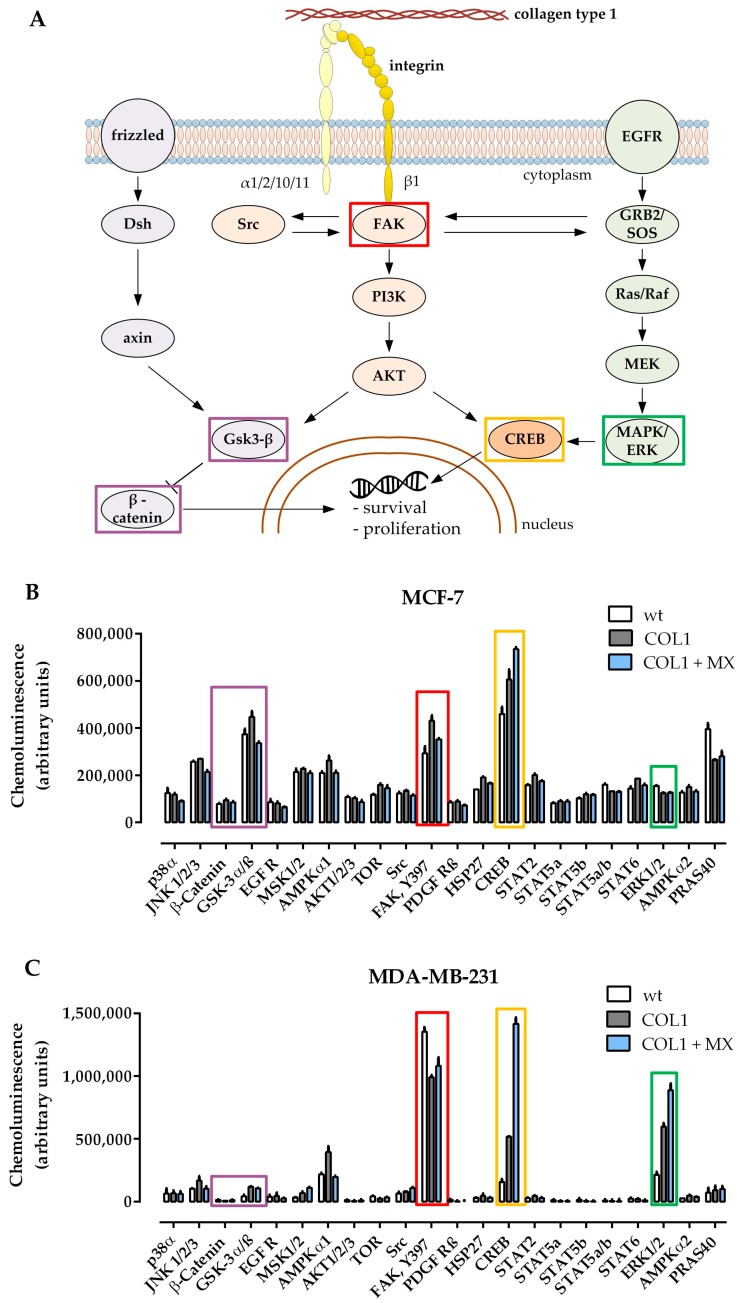
β1-Integrin-related signaling pathways and their deregulation by COL1 binding. (**A**) Schematic illustration of three potential signaling pathways induced by β1-integrin binding to COL1 as potential contribution to increased cell survival. Wnt, FAK/PI3K/AKT, and MAPK pathways are highlighted in purple, red and green, respectively. (**B**) Data set of a human proteome profiler kinase activity array of MCF-7 cells and (**C**) of MDA-MB-231 cells cultivated on plain surfaces (‘wt’); cultivated on COL1 (‘COL1’) or treated with the EC_50_ values of MX when cultivated on COL1 (‘COL1 + MX’). Each sample consists of a cell lysate, standardized to 200 µg protein per membrane. Data refer to a deregulation of FAK (red), CREB (orange), ERK1/2 (green), β-catenin and GSK-3α/β (both purple). Their roles in the respective signaling pathways are indicated in (**A**). COL1: Collagen type 1, MX: Mitoxantrone.

To obtain a first insight of whether and how the potential β1-integrin signaling pathways (schematically depicted in Figure 1A) were involved, a human kinase proteome profiler array was applied comparing the kinase activity status of untreated MCF-7 (Figure 1B) or MDA-MB-231 (Figure 1C) cells with COL1 cultivated cells and cells exposed to combined treatment with MX (EC_50_) cultivated on COL1.

One of the most remarkable shifts in kinase phosphorylation of MCF-7 cells is the upregulation of FAK by COL1. FAK represents a well-known key molecule in the β1-integrin binding process and is an upstream component of the PI3K/AKT pathway. In presence of MX, the COL1 effect on FAK activity is slightly diminished. In contrast, MDA-MB-231 cells showing a higher base FAK activity display a slight reduction of pFAK by binding to COL1.

Gsk-3α/β displays a higher phosphorylation state after COL1 cultivation, but not in presence of MX in case of MCF-7. Since Gsk-3β is a crucial component of the Wnt signaling pathway, which can also be affected by the PI3K/AKT trail, Wnt signaling pathway should be considered a potential candidate mediating a survival benefit of MCF-7 cultivated on COL1. Although Gsk-3α/β is slightly elevated in MDA-MB-231 upon the treatments, its protein amount is on a neglectable level, such as β-catenin. Therefore we precluded Wnt pathway as a dominant pathway in MDA-MB-231.

In contrast to MCF-7, MDA-MB-231 cells react to COL1 or COL1 + MX by increasing pERK1/2 (=MAPK) up to 3-fold or 4-fold levels respectively, providing an entirely different survival strategy than MCF-7.

Furthermore, both cell lines display that CREB is strongly activated in presence of COL1 and even more in the combined treatment of COL1 and MX. In case of MDA-MB-231 the activated pCREB is increased from 3-fold (COL1) up to 10-fold (COL1 + MX). CREB serves as a cellular transcription factor and therefore is one of the final targets of MAPK signaling inside the cytosol, which is also accessible by FAK and PI3K/AKT [26].

In summary, three potential β1-integrin related signaling pathways offer a strategic target to tackle survival activity induced by COL1 binding. MDA-MB-231 cells display a high MAPK activity, whereas FAK/PI3K/AKT pathway seems to be the dominant pathway in MCF-7 cells, possibly in combination with Wnt.

### 2.2. The Impact of Wnt Signaling on COL1 Induced Resistance Formation in MCF-7 Cells

Related to the moderate increase in phosphorylation of Gsk-3α/β at serine 9 (Figure 1B), of which Gsk-3β is associated with a deactivation of this kinase and consequently an enhancement of the Wnt signaling pathway [27], we initially focused on Wnt signaling in MCF-7 cells and the impact of COL1 and cytotoxic treatments. Therefore, we performed a flow cytometric detection of selected Wnt pathway components. On the one hand, β-catenin is considered a key component of this pathway which, upon translocation into the nucleus activates transcription by binding to transcription factors of the T-cell factor and the lymphoid enhancer factor (TCF/LEF) family [19]. On the other hand, we investigated the structure protein axin that forms the Wnt specific destruction complex by binding and stabilizing Gsk-3β and other factors to antagonize β-catenin activity. To further focus on integrin-related cell activities, manganese II (Mn(II)) as an allosteric activator of integrins was applied providing an alternative mode of action other than COL1-ligand-binding to MCF-7 cells.

**Figure 2:**
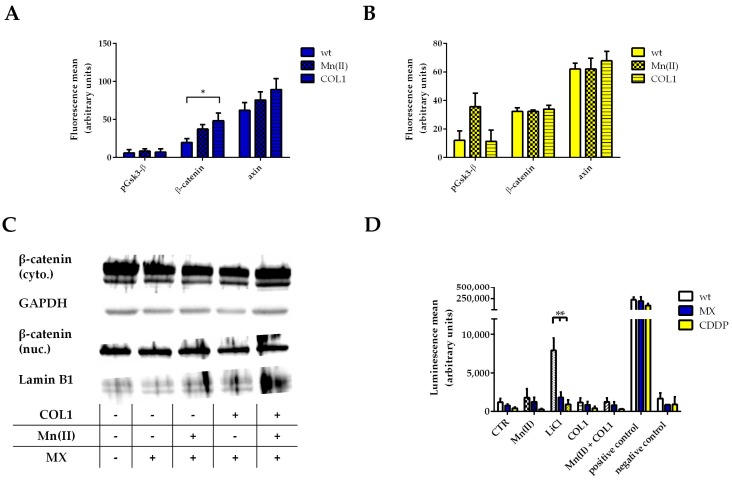
Investigation of a potential involvement of Wnt signaling in MCF-7 resistance formation. Flow cytometric detection of Wnt components in MCF-7 cells treated with EC_50_ of MX (**A**) or CDDP (**B**) influenced by COL1 or integrin activation by Mn(II). (**C**) Western blot data of MCF-7 cells treated in the same manner as (**A**) confirm that β-catenin levels remain unchanged inside the cytosol (cyto.) and the nucleus (nuc.). (**D**) Detection of Wnt activity in MCF-7 cells by TOP-flash assay. Data indicate that Wnt activity is not affected by COL1 or Mn(II) in response to MX or CDDP. Presenting the mean of at least *n* = 3 (±SEM), asterisks indicate statistical significance: * *p* < 0.05, ** *p* < 0.01.

Treatment of MCF-7 cells with an EC_50_ of MX resulted in a slight increase of β-catenin upon COL1 or Mn(II) incubation, however, axin is also slightly increased indicating no remarkable activation of the Wnt pathway. In agreement to that, phosphorylation of Gsk-3β at serine 9 displays no detectable deregulation (Figure 2A). In case of CDDP treatment (EC_50_) of MCF-7 cells, similar findings were obtained (Figure 2B). Only Mn(II) induced a certain increase in phosphorylation of Gsk-3β but this did not influence β-catenin levels. An accumulation of β-catenin in the nucleus could also be excluded (Figure 2C).

To further focus on Wnt signaling activity of MCF-7 cells, we transiently transfected the cells with a reporter plasmid containing the TCF/LEF promotor region coupled with a firefly luciferase gene (TOP-flash assay). LiCl served as a positive control, since its capacity to inhibit Gsk-3β is often used in those types of assays. The LiCl positive controls showed high luminescence values proving that Wnt signaling can be activated in MCF-7 cells as well as the positive control firefly luciferase. However, the luminescence data clearly exclude an upregulation of Wnt activity in all approaches (Figure 2D). Neither the cell cultivation on COL1, nor Mn(II) alone nor in combination with COL1 induced a higher transcriptional activity in response to MX or CDDP. Summarizing, the Wnt signaling pathway is not involved in the observed higher resistance of MCF-7 cells against a CDDP or MX treatment and thus does not appear as a promising target to sensitize cells in presence of their microenvironment.

Since the proteome profiler array displayed no change or relevant activity in Gsk-3α/β and β-catenin in MDA-MB-231 cells, we precluded the Wnt pathway. Nevertheless we investigated the levels of β-catenin upon MX and COL1 by Western blot, showing no differences (Figure S1). Consequently, considering the direct functional linkage between FAK and integrins and our proteome profiler data, we proceeded investigating the FAK/PI3K/AKT pathway.

### 2.3. FAK/PI3K/AKT Pathway as Potential Targets for MCF-7 and MDA-MB-231 Sensitization

FAK is a key component of integrin signaling, which upon recruitment of the Src kinase induces a signal transduction e.g., via the PI3K and AKT pathway. This pathway has been shown to contribute to tumor malignancy [28]. To obtain an insight whether these kinases were deregulated in the MCF-7 cells upon COL1 binding as well as Mn(II) activation of integrins in absence or presence of MX, we performed Western blot investigations comparing the non-activated form of the kinases with the phosphorylated, i.e., activated subtypes.

**Figure 3:**
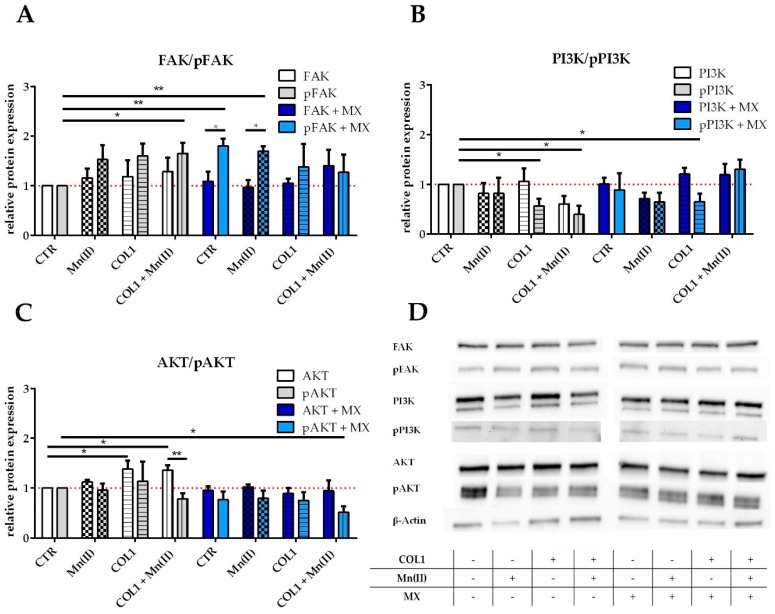
Western blot data of FAK/PI3K/AKT pathway components in MCF-7 cells and their deregulation by integrin activation and MX cytotoxic treatment. Protein levels of (**A**) FAK and pFAK; (**B**) PI3K and pPI3K; (**C**) AKT and pAKT are displayed normalized to total protein stainfree analysis and in relation to untreated MCF-7 cells as control (CTR, red line for comparison). The samples were treated in-between activation by Mn(II), COL1 or combined Mn(II) and COL1 in absence of MX (grey) or presence of EC_50_ MX (blue). (**D**) Shown is a representative Western blot, but all experiments were conducted in at least *n* = 3 (±SEM), asterisks indicate statistical significance: * *p* < 0.05, ** *p* < 0.01.

FAK is clearly upregulated by the triggers COL1 or Mn(II) and slightly in presence of MX (Figure 3A,D). In addition, the tyrosine 397-phosphorylated FAK (pFAK), indicating the active conformation of the enzyme, displays an upregulation up to 1.5 fold by integrin activation in absence of MX, but pFAK accumulates even significantly more in presence of MX. This could be an indicator of a cell defense strategy against the cytotoxic stress upon integrin stimulation and qualifies FAK as a potential target for sensitization attempts.

PI3K displays unchanged levels of protein when MCF-7 cells were activated by Mn(II) or COL1, but the addition of MX appears to have an increasing effect on PI3K levels (Figure 3B,D). The phosphorylated form of PI3K is decreased in presence of MX or COL1 and Mn(II) incubated cells.

The downstream component AKT in its non-phosphorylated state displays a certain increase in presence of integrin stimuli especially by COL1 (Figure 3C,D). The phosphorylated AKT (pAKT) shows besides slightly increased levels in COL1 binding a downregulation in presence of MX.

Based on these findings, we assume that the FAK/PI3K/AKT pathway is partially deregulated upon integrin activation by COL1 binding, which likely contributes to a gain in survival capabilities under a MX treatment and therefore appears as promising target for sensitization.

In order to investigate whether the qualitative findings of signaling deregulation in the FAK/PI3K/AKT pathway obtained by western blot are reflected by functional sensitization studies (MTT assay), we inhibited FAK and PI3K to evaluate a potential impact on sensitization. We selected FAK inhibitor 14 (FAK14, 1 µM) and the PI3K inhibitor BEZ235 (1 nM) and studied the impact on sensitivity against MX and CDDP. Both inhibitors display no intrinsic cytotoxicity at the specified concentrations (Figure S2A,B).

**Figure 4:**
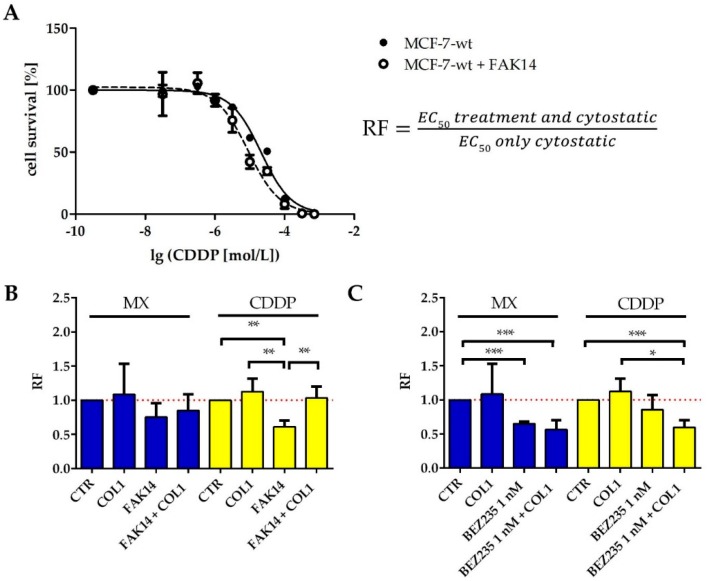
Inhibition of FAK and PI3K and the impact on MCF-7 sensitivity to MX and CDDP cytotoxicity. (**A**) Exemplary data of MTT results to clarify calculation of the resistance factor (RF) as ratio of EC_50_ values of treated (hollow circle) vs. untreated (full circle) control MCF-7 cells (CTR). The RF confirms that (**B**) Inhibition of FAK in MCF-7 cells by FAK14 at 1 µM increases sensitivity against MX (blue) and CDDP (yellow). (**C**) Inhibition of PI3K by BEZ235 at 1 nM significantly sensitizes the MCF-7 cells to MX and CDDP precluding the resistance fostering effect by COL1 observed in absence of BEZ235. Data are presented as EC_50_ ratios of treated cells vs. untreated cells (RF) in each measurement as means of at least *n* = 3 (±SEM), asterisks indicate statistical significance: * *p* < 0.05; ** *p* < 0.01; *** *p* < 0.001.

FAK inhibition clearly sensitizes the MCF-7 cells for MX and CDDP cytotoxicity (Figure 4B, blue), indicated by lower EC_50_ values than those of the sole cytostatic treatment. The ratio of EC_50_ values of treated cells vs. untreated control, referred to as resistance factor (RF, illustrated in Figure 4A) is consequently below 1, indicating a cell sensitization. Notably, even in presence of COL1, FAK14 sensitizes the MCF-7 cells for MX toxicity. In case of CDDP (Figure 4B yellow), FAK inhibition also induces a significant sensitization, which is of minor extent in presence of COL1.

The inhibition of PI3K also appears promising, considering the data in Figure 4C. BEZ235 sensitizes the MCF-7 cells significantly for MX cytotoxicity, COL1 induced activation of the cells is seemingly antagonized and cell sensitivity is further increased. Similar findings were evident in case of CDDP shown by the reversal of the COL1 induced resistance via BEZ235.

Although the proteome profiler array provided no evidence for upregulated FAK/PI3K/AKT in MDA-MB-231 cells, and FAK was even being decreased upon integrin stimulation, we performed functional sensitization studies (Figure S3) using the same inhibitors at non-toxic concentrations (Figure S2C,D). The inhibition of FAK displayed a slight sensitization, whereas PI3K inhibition was without effect on increased survival. While FAK is necessarily connected to β1-integrin-mediated COL1-binding and also connected to MAPK pathway, this effects support the involvement of MAPK pathway.

Taken together, these data underline the key role of the FAK/PI3K/AKT signaling pathway for MCF-7 cell survival under MX or CDDP cytotoxic stress in presence of the extracellular matrix component COL1. Consequently, FAK and PI3K appear as promising targets in MCF-7 cells to interfere with CAM-DR and suggest a proof of concept experiment by a β1-integrin knockdown.

### 2.4. β1-Integrin Knockdown in MCF-7 Cells and Consequences for Sensitization

**Figure 5:**
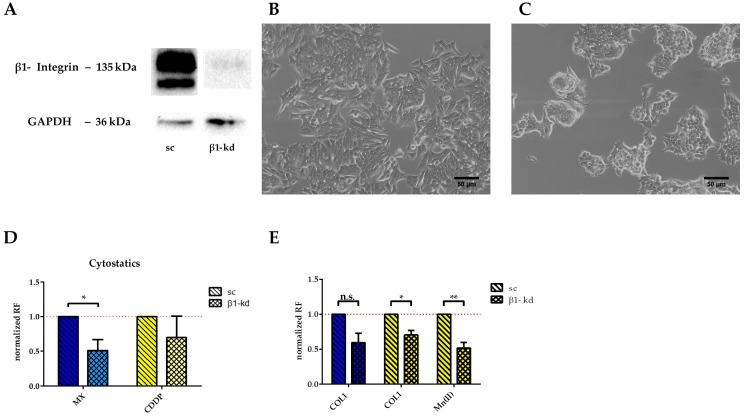
β1-Integrin knockdown in MCF-7 cells. (**A**) Western blot data confirm the almost complete deletion of β1-integrin in the knockdown cells compared to the scrambled vector control. (**B**) The scrambled control MCF-7-sc cells display a typical spreading at a COL1 surface, which is completely different for the MCF-7-β1-kd cells (**C**), Scale bar:50 µm. (**D**) The MCF-7-β1-kd cells display a higher sensitivity against MX and CDDP cytotoxicity normalized to the scrambled control cells. (**E**) Upon CDDP treatment, the MCF-7-β1-kd cells show a lower response to COL1 binding with respect to EC_50_ compared to scrambled control and are less responsive to Mn(II). Data are means of at least *n* = 3 (±SEM), asterisks indicate statistical significance: * *p* < 0.05; ** *p* < 0.01; *** *p* < 0.001.

To confirm that the higher resistance of MCF-7 cells against MX and CDDP induced cytotoxicity is dependent on β1-integrins, we performed a knockdown using lentiviral vectors. As indicated in Figure 5A, the knockdown cells (MCF-7-β1-kd) display minimal to no remaining β1-integrin subunit in the Western blot analysis compared to the control cells transfected with a lentiviral vector coding for scrambled shRNA (MCF-7-sc). As functional evidence of the successful knockdown approach, cell growth on COL1 is illustrated comparing MCF-7-β1-kd and MCF-7-sc cells. While the MCF-7-sc cells display a growth pattern behavior typical for MCF-7 cells on COL1 (Figure 5B), the knockdown cells avoid a spreading at the COL1 surface due to the β1-integrin deficiency and tend to grow in cellular clusters (Figure 5C).

The knockdown cells display a higher sensitivity towards MX and CDDP cytotoxicity (Figure 5D), taking the scrambled cells as control and considering the resistance factor as ratio of EC_50_ of knockdown cells and scrambled cells. It becomes evident that, without any further cell activation, the MCF-7-β1-kd cells are significantly more sensitive to MX. Considering the CDDP treatment with respect to integrin activation of the cells, the knockdown cells are slightly more sensitive in contact with COL1. Furthermore, Mn(II) activation of integrins is also unable to stimulate the knockdown cells, compared to the scrambled control cells (Figure 5E). In case of MX, the MCF-7-β1-kd cells were less resistant when cultivated on COL1 due to absence of β1-integrin compared to the scrambled control. However, the integrin activation by Mn(II) cannot be performed in presence of MX since anthracyclines and probably anthracycline-like drugs as well, are known to form complexes with divalent cations [29]. Instead, we checked the efficiency of FAK inhibition upon β1 stimulation by COL1 binding. Whereas the EC_50_ values of the knockdown cells is almost unaffected by FAK inhibition (0.1 µM -COL-1 and 0.14 µM -COL1 + FAK), the sc cells were sensitized, displaying values of 0.18 µM (COL1) to 0.06 µM (COL1 + FAK14). This illustrates the importance of FAK as a pharmacological target in sensitization strategies of β1-integrin expressing cancer cells. Nevertheless it is likely that even in absence of β1-integrins FAK maintains a crucial role beside the FAK/PI3K/AKT pathway inducing a crosstalk signaling to, e.g., the MAPK pathway, as indicated in Figure 1A.

### 2.5. MAPK-Pathway Components as Potential Target for Sensitization Strategies in MDA-MB-231

The significantly upregulated levels of pERK1/2 (Figure 1C) clearly identify the MAPK pathway in its dominance in EGFR expressing MDA-MB-231 cells. Furthermore, the phosphorylated form of the cellular transcription factor cAMP response element-binding protein (pCREB), a downstream component mainly of the MAPK, but also of PI3K/AKT pathway, appeared as the most distinctively deregulated kinase in MDA-MB-231 cells, when treated with COL1 and MX (Figure 1C). MAPK pathway is generally considered a key regulator of cell growth and proliferation, triggered by growth factor receptors and/or stimulated by cell adhesion, e.g., via FAK [30].

**Figure 6:**
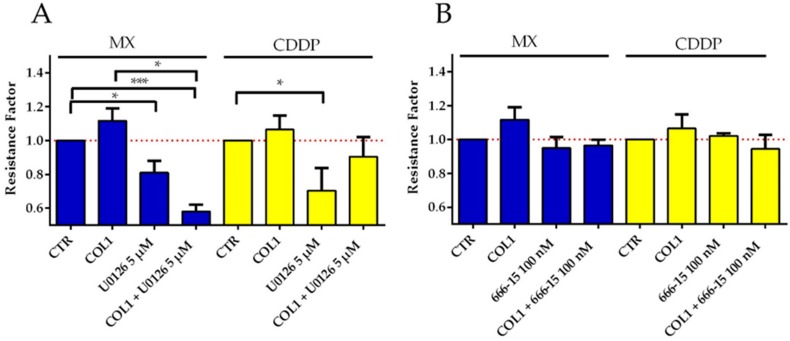
Inhibition of MAPK pathway in MDA-MB-231 cells as a sensitizing strategy. (**A**) Inhibition of MEK and concurrently downstream molecule ERK in MDA-MB-231 cells by U0126 at 5 µM increases sensitivity against MX (blue) and CDDP (yellow). (**B**) Inhibition of CREB by 666-15 at 100 nM slightly sensitizes the MDA-MB-231 cells to MX and CDDP cytotoxicity, but not to the extent of U0126. Data are presented as EC_50_ ratios of treated cells vs. untreated cells (RF) in each measurement as means of at least *n* = 3 (±SEM), asterisks indicate statistical significance: * *p* < 0.05; ** *p* < 0.01; *** *p* < 0.001.

Inhibition of MEK1/2 and thereby the downstream component ERK1/2 by U0126 (5 µM) increased cytotoxic activity of MX and CDDP significantly (Figure 6A). In case of MX, this effect is even amplified in combination with COL1 reflecting the further increased levels of pERK1/2 presented in Figure 1C. Although CREB is highly deregulated in MDA-MB-231 cells, its inhibitor 666-15 (100 nM) induces only a slight sensitization for MX and CDDP cytotoxicity (Figure 6B). Both inhibitors were shown to possess no intrinsic cytotoxicity at the applied concentration (Figure S4A,B).

### 2.6. MAPK Pathway as ‘Secondary Target’ for Sensitization Strategies in β1-Integrin Deficient MCF-7 Cells

Since pCREB is elevated in treated MCF-7 cells without showing an increased ERK activity (Figure 1B), we evaluated the impact of the MAPK pathway in MCF-7 cells, too. We focused on the key components of this pathway by Western blot, taking into account the integrin activating triggers COL1 binding to MCF-7 cells and Mn(II) in absence or presence of a MX treatment at EC_50_.

**Figure 7:**
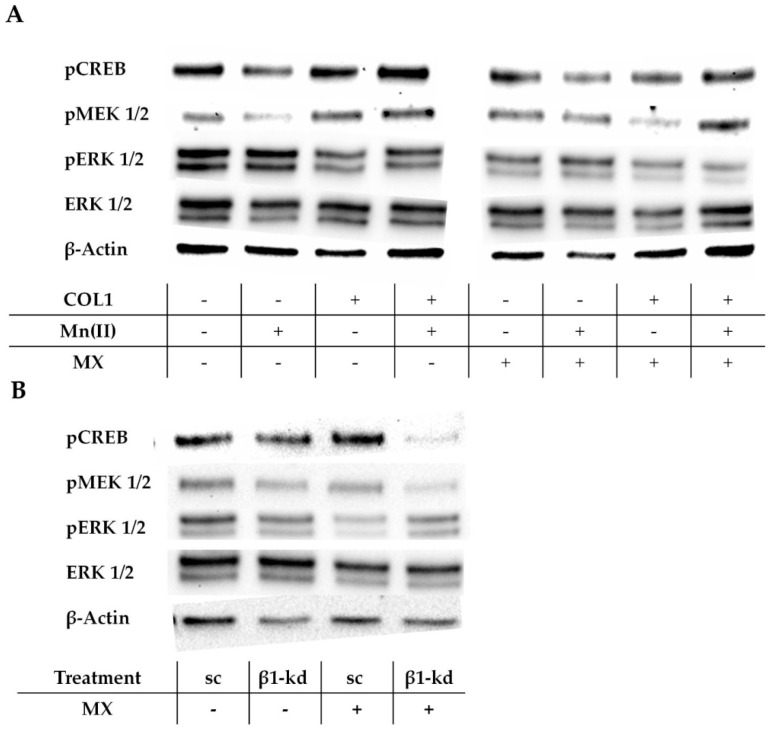
The role of MAPK pathway in MCF-7 cells to regulate the sensitivity to MX cytotoxicity. (**A**) Representative western blot data (*n* = 3) of the MAPK pathway components in MCF-7 cells in relation to integrin activation by COL1 or Mn(II) or combination thereof in absence of MX (left) or in presence of EC_50_ of MX (right). (**B**) Deregulation of the indicated MAPK pathway components in MCF-7 scrambled control (sc) or MCF-7-β1-kd cells (β1-kd), illustrated by a representative western blot (*n* = 3).

In absence of a MX treatment, pCREB is upregulated by COL1 (Figure 7A, left), which confirms the proteome profiler data indicated above. The phosphorylated MAPK/ERK kinase (pMEK) appears also more intensified, while the ERK and pERK were not deregulated by integrin activation. When cells were treated with MX, the levels of pCREB, pMEK, and pERK were attenuated in the COL1 activated cells (Figure 7A, right). This indicates the involvement of the MAPK pathway to affect the MX activity in the MCF-7 cells in presence of COL1 and draws the attention to pCREB, pMEK and pERK as potential targets for sensitization strategies.

To further evaluate the role of the MAPK pathway and its impact on cell survival in relation to β1-integrin activity, we also checked these pathway components in the MCF-7-β1-kd cells in comparison to MCF-7-sc cells (Figure 7B; Figure S5A–D). It is clearly evident that even upon a dominant knockdown of β1-integrin, ERK and pCREB mainly show a similar protein expression, whereas pMEK and pERK are mitigated. Notably, when treated with an EC_50_ of MX the sc and kd cells differ in their deregulation of phosphorylation. While the MCF-7-sc cells decrease their amount of pERK, which is consistent with the findings in Figure 7A, the MCF-7-β1-kd cells seems to be unaffected by MX regarding pERK. Furthermore pMEK is slightly reduced in MX treated MCF-7-sc cells, whereas MCF-7-β1-kd cells show no difference. Interestingly, pCREB shows the most striking deregulation. In MX-treated MCF-7-sc cells, pCREB is slightly upregulated, while the MCF-7-β1-kd cells show an evidently decreased expression. This illustrates a specific susceptibility of pCREB as signaling component reacting in response to MX and highlights this kinase as an attractive target to modify the cellular response to MX. It appears likely that the role of the MAPK pathway and especially pCREB as target are more pronounced when the classical integrin signaling routes, such as FAK/PI3K/AKT were diminished beforehand by a functional β1-integrin knockdown.

**Figure 8:**
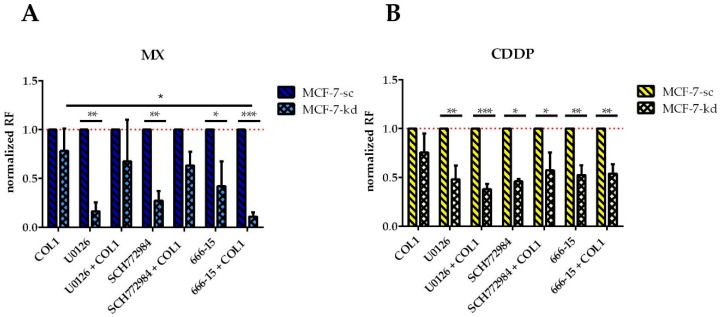
MTT based cytotoxicity studies of (**A**) MX or (**B**) CDDP in MCF7-sc and MCF-7-β1-kd cells in dependence on COL1 binding and the impact of the MEK inhibitor U0126 at 5 µM, the ERK inhibitor SCH772984 at 250 nM, the CREB inhibitor 666-15 at 100 nM concentrations. Data are presented as EC_50_ ratios of treated cells vs. untreated cells (RF) normalized to sc cells. Data are means of at least *n* = 3 (±SEM), asterisks indicate statistical significance: * *p* < 0.05; ** *p* < 0.01; *** *p* < 0.001.

To prove whether targeting of the MAPK pathway components could sensitize the MCF-7 cells either in presence of β1-integrins as ‘secondary’ targets or in their absence as ‘primary’ targets, we investigated the impact of an inhibition of ERK by SCH772984, of MEK by U0126 and of CREB by 666-15 on MX and CDDP cytotoxicity, either in presence or absence of COL1 (Figure 8). Affected sensitivity is again presented as EC_50_ ratio of treated cells to the untreated control cells, expressed as ‘resistance factor’. Because the indicated MAPK inhibitors did not sensitize sc cells, we normalized the sensitivity data of the kd cells to the respective sc controls. All compounds were shown to possess no, or a negligible intrinsic cytotoxicity at the applied concentration (Figure S4C–E).

Concerning ERK, the inhibitor U0126 has a significantly increased sensitizing effect on MCF-7-β1-kd cells against either MX (Figure 8A) or CDDP (Figure 8B). This effect is partially antagonized by COL1 in case of MX, but in case of CDDP it is even strengthened. Inhibition of ERK by SCH772984 seems comparable to inhibition by U0126 only differing in case of CDDP, where the combination of COL1 and SCH772984 displays a slightly less sensitizing effect.

The inhibition of CREB by 666-15 proves to be more effective than ERK and MEK inhibition, indicated by a significant sensitizing effect for MX in β1-integrin deficient cells, which is even highly significant in combination with COL1. This is also evident in CDDP treatment, but to a lesser extent and cultivation on COL1 has no influence.

Consequently, the MX cytotoxicity data support the assumption of CREB as attractive ‘secondary’ target in absence of β1-integrins. While CREB inhibition has no effect in the β1-integrin competent MCF-7-sc cells (data not shown), it can significantly sensitize the MCF-7-β1-kd cells in presence of COL1. These data highlight CREB, among the other components of this pathway as a promising target similar to the Western blot data in Figure 6B, which show a massive CREB deregulation under MX treatment in the knockdown cells.

Concluding, these data confirm the role of the MAPK pathway to control the sensitivity of the MCF-7 cells to respond to CDDP and MX cytotoxicity. It becomes evident that this pathway appears dominant when β1-integrin activity is attenuated. One might assume that MAPK is activated subsequently via a crosstalk to FAK, or via an alternative external trigger to mediate a matrix binding effect into the cell directly via the MAPK pathway.

## 3. Discussion

Resistance formation of tumors against a cytotoxic treatment regime appears as the dominant obstacle in the clinical treatment of cancer patients [31]. Tumor cells make use of versatile molecular mechanisms to circumvent a pharmacological induction of apoptosis. However, there is presently no therapeutic sensitization approach for an interference with resistance formation of tumors available or in process of approval. In search of novel targets to sensitize breast cancer cells against the guideline-based drugs MX and CDDP, we focused on CAM-DR. This phenomenon is known as a non-genetically based survival strategy of tumors triggered by cell binding to components of the microenvironment. Although the acquired resistance is only moderate, CAM-DR is considered as essential prerequisite to confer a prolonged survival of tumor cells towards antineoplastic agents and thus favors the acquisition of genetic drug resistance mechanisms [32]. CAM-DR has attracted the primary attention in the treatment of multiple myeloma, but since then has also been accepted for its relevance in various solid tumors [5,6]. Therefore, therapeutic interference with CAM-DR appears promising to antagonize resistance as an early onset mechanism, relevant for different other types of resistance formation. However, the underlying mechanisms of CAM-DR are still not fully understood.

We were able to simulate CAM-DR by showing a decrease in sensitivity of human MCF-7 and MDA-MB-231 breast cancer cells to MX or CDDP cytotoxicity when cells were cultivated in presence of COL1. COL1 is among fibronectin, laminin and vitronectin by far the most abundant protein of breast tissue and mainly influences breast density [33]. The importance of COL1 is clinically reflected by a 2–5 fold higher risk of getting breast cancer in patients with a high density of breast tissue [34,35,36] and also with a more negative outcome due to its role in metastasis processes [37]. Therefore interfering with COL1 appears as a promising strategy in breast cancer, although the role of the other components of ECM e.g., proliferation increasing properties of fibronectin have to be kept in mind and are maybe more relevant for other cancer entities [38]. The changes in EC_50_ values are not as high as in genetically resistant cell lines, based on the epigenetic nature of CAM-DR. Nevertheless the presence of COL1 provides a more physiological insight of the cells’ behavior. β1-Integrin as preferred cellular binding partner of COL1 was considered a key molecule to induce a loss in sensitivity in this cellular model, and its important role was confirmed by a knockdown approach. This approach is consistent with other findings, which identified β1-integrin as a bad prognostic marker for breast cancer associated with a worse overall and disease free survival of the patients [39] or as a target to improve radiotherapy of breast cancer [40]. In these terms, antibody mediated β1-integrin inhibition increased the sensitivity of glioblastoma xenografts to an anti-angiogenic treatment [41]. However, a therapeutic blockade of β1-integrin appears complex in light of the ubiquitous expression and function of this integrin subtype in the body. Clinical approaches of e.g., the α5β1-integrin by the antibody volociximab in other terms of oncology have shown insufficient efficacy for further development [42].

Consequently, an interference with β1-integrin signaling axes by small molecule inhibitors, probably in combination with drug carriers for site-specific targeting approaches of tumors, appears to have better prospects for sensitization. Among the three most deregulated pathways that we identified upon COL1 cell binding under MX treatment, Wnt signaling pathway could be excluded to significantly contribute to a loss in CDDP or MX sensitivity and was therefore excluded as potential target. Instead, we found the FAK/PI3K/AKT axis to be active in MCF-7 cells and MAPK pathway to be dominant in the MDA-MB-231 cell line, possibly correlated to its EGFR expression.

FAK/PI3K/AKT pathway was shown to be crucially involved in sensitivity loss and consequently, FAK was identified as potential targets for a sensitization of MCF-7 and MDA-MB-231 cells, while inhibition of PI3K only affected MCF-7. FAK has been considered a target for several antitumor approaches, such as blocking breast cancer growth and metastasis [43]. However, the relation between FAK and therapy resistant tumors is less apparent. A doxorubicin-resistant subtype of MCF-7 cells has been shown to be associated with an upregulated FAK [44]. Furthermore, FAK/Scr activation has been related to a trastuzumab resistance in HER-2 overexpressing cells [45] or with a gemcitabine resistance in triple negative breast cancer cells. We can show that FAK inhibition antagonizes the COL1 effect of MCF-7 and MDA-MB-231 cells and thus sensitizes them for MX and CDDP cytotoxicity.

Following this pathway downstream, PI3K is regarded as a critical component in oncogenesis and resistance formation in general, and also in terms of breast cancer. There are multiple therapeutic approaches to evaluate the role of PI3K in its isoform selectivity as target for antitumor activities [46]. In breast cancer, PI3K was mainly considered a key point to affect resistance against HER-2 targeted therapies [47]. For instance, inhibition of PI3Kα by alpelisib was shown to attenuate the resistance against trastuzumab and taxanes in HER-2 positive breast cancer [48]. Apart from that, PI3K was shown as target to sensitize breast cancer cells for adriamycin toxicity [49] and the combined PI3K/mTOR inhibitor BEZ235, that we also used here, was shown to sensitize genetically modified CDDP resistant breast cancer cells for cytotoxicity in a recent study [50]. In our study, BEZ235 displayed significant effects to antagonize the COL1 induced sensitivity loss against MX and CDDP in MCF-7 cells, but not in MDA-MB-231. This selective involvement of FAK and not PI3K proves that MAPK pathway is dominant in MDA-MB-231. Thus, PI3K appeared as the most promising target to sensitize the MCF-7 cells in the present approach. Although inhibition of PI3K is considered in its potency as target for several aspects in oncology, cells react differently and dynamically to inhibitory approaches and can drive resistance or tumorigenicity via rewiring of signaling pathways [51]. An active crosstalk of the PI3K/AKT pathway with MAPK signaling route is known [52] and becomes also evident by our findings. We can show that components of the MAPK pathway are obviously not directly engaged in the sensitivity loss of the MCF-7 cells but in EGFR expressing MDA-MB-231. Notably, in the MCF-7-β1-integrin knockdown cells, inhibition of MEK, and even more ERK and CREB is efficient to overcome COL1 induced CAM-DR and foster cell sensitization. The CREB inhibitor 666-15 is able to significantly increase the sensitivity against MX and CDDP when β1-integrin deficient cells were cultured on COL1. Considering the intracellular signaling, the phosphorylation of CREB as a reaction to cytostatic treatment seems to be dependent on the presence of β1-integrins, since MCF-7-sc cells reacted by upregulating pCREB, which was also found in HMESCO and H2373 cells under a doxorubicin treatment [53]. On the contrary MCF-7-β1-kd cells strikingly downregulate this target under MX treatment. Specifically inhibiting CREB as a target for sensitization strategies is a novel approach.

It is elusive why MAPK becomes more engaged and targetable when β1-integrins are dominantly knocked down. Apart from the dynamic shifting of signaling routes, mentioned above, other cellular binding partners of COL1 should be considered. The discoidin domain receptor DDR1 is considered more and more as a molecule that transfers extracellular binding events into intracellular signaling cascades. DDR1 is a binding partner of COL1, and MCF-7 cells cultivated on COL1 have been described to be attenuated in their apoptotic response by DDR1 acting via ERK1/2 [54]. DDR1 is found to be connected to several intracellular pathways upon binding to COL1, but MAPK pathway appears to be dominant [55,56]. Nevertheless, a possible crosstalk between DDR1 and PI3K/AKT has also been described [57,58]. In light of these considerations, our data lead to the following conclusions.

β1-Integrin-mediated COL1-binding transmits its desensitizing signaling dominantly via the FAK/PI3K/AKT route in MCF-7 cells and via MAPK in MDA-MB-231 cells, possibly in a synergetic way with DDR1. These differences in intracellular signaling are possibly correlated to the cells’ different reaction to COL1 on a biomechanical level. Consequently, ERK appears as a primary target in MDA-MB-231 cells, while FAK and PI3K display the most favorable targets for a sensitization of cells in MCF-7 cells. The direct activation of the MAPK pathway in MCF-7 via FAK might occur, but does not directly contribute to the observed loss in sensitivity. However, when the dominant β1-integrin axis is disturbed, MAPK takes over to drive the resistance, probably activated by DDR1 interacting with COL1. This explains that components of the MAPK pathway appear as attractive ‘secondary’ target. We outlined such an approach for CREB in the β1-integrin knockdown cells. From a therapeutic point of view, a combined treatment with two selected inhibitors cutting primary and secondary signaling routes would appear most promising, which should be investigated in future studies.

All in all, these findings illustrate the potential of a therapeutic interference with intracellular signaling pathways, differing in the various tumor entities, to disrupt the processes of CAM-DR and therefore deprive the cells of the suitable conditions to generate genetically based resistance mechanisms at an early onset point.

## 4. Materials and Methods

### 4.1. Cell Culture and Reagents

The human MCF-7 and MDA-MB-231 breast cancer cell line was cultivated in DMEM supplemented with penicillin (10 IU/mL), streptomycin (100 μg/mL), L-glutamine (2 mM) and 10% FBS (plus 1% sodium pyruvate in case of MDA-MB-231), in a humidified atmosphere at 37 °C containing 5% CO_2_. Cells were detached using a solution of EDTA (0.2 g/L EDTA × 4 Na) for 10 min at 37 °C. All reagents were from Thermo Fisher Scientific Inc. (Waltham, MA, USA). Cell identity was evaluated using a STR profile analysis. MX was obtained from Hexal AG, Holzkirchen, Germany and CDDP from Sigma-Aldrich Chemie GmbH, Taufkirchen, Germany.

96-well plates were coated with COL1 (Corning, Thermo Fisher Scientific Inc.) at a density of 10 μg/cm^2^, according to the manufacturers’ protocol. For flow cytometry and western blot experiments, collagen coated cell flasks were used (Sarstedt AG & Co, Nümbrecht, Germany). Manganese(II)chloride solution (1 mM) was added to the cells for 5 min prior to the incubation with cytostatics, in order to allosterically activate integrins [59]. BEZ235 (dactolisib), a dual pan-PI3K/mTOR inhibitor with imidazole [4,5-c]quinolone structures was obtained from Selleck Chemicals (Houston, TX, USA). Cells were treated with 1 nM of BEZ235, for 6 h, prior to the start of the MTT assay. For the inhibition of FAK phosphorylation at Y397, a FAK inhibitor 14 (Biomol, Hamburg, Germany) was applied to the cells at a concentration of 1 µM, 4 h before conducting MTT experiments [60]. Inhibitors of the MAPK signaling pathway were investigated including MEK inhibitor U0126 (Selleck Chemicals) at a concentration of 5 µM, ERK1/2 inhibitor SCH772984 (Hycultec, Beutelsbach, Germany) at 250 nM and CREB inhibitor 666-15 (Hycultec) at 100 nM. The indicated inhibitors were added to the cells 4 h prior to cytostatic treatment.

### 4.2. MTT Assay

Cell viability assay using MTT 3-(4,5-dimethylthiazol-2-yl)-2,5-diphenyltetrazolium bromide (BioChemica, Applichem GmbH, Darmstadt, Germany) was conducted as described [61]. 2000 MCF-7 or 4000 MDA-MB-231 cells were seeded in triplicates at a total volume of 100 µL per well of 96-well plates (Sarstedt AG) either coated with COL1 or left uncoated. The next day, cells were supplemented with 1 mM MnCl_2_ (Merck KGaA, Darmstadt, Germany) not the drug company) as well as a dilution series of the cytostatics CDDP (10^−3.3^ to 10^−7.5^ M), or MX (10^−4.5^ to 10^−8.5^ M). In case of MnCl_2_, the medium was renewed after an incubation of 5 min to exclude cytotoxicity. After an incubation of 72 h, a MTT solution (20 µL, 5 mg/mL) was added for 1 h at 37 °C and 5% CO_2_. After removing the supernatant, formazan was solubilized in 200 µL of DMSO (Carl Roth GmbH, Karlsruhe, Germany). Plates were analyzed using a plate-reader (Thermo Multiscan EX, Thermo, Schwerte, Germany) at 570 nm, with background subtraction at 690 nm. Data were normalized to DPBS as 100% viability and the bottom value as 0% viability and evaluated by a nonlinear regression.

### 4.3. Flow Cytometry

Flow cytometry experiments were conducted according to the protocol of Piva et al. [20]. Cells were incubated for 2 h with rabbit anti-β-catenin, anti-axin (GeneTex Inc., Irvine, CA, USA) and goat anti-pGsk-3β (Ser9) antibodies. Donkey anti-rabbit IgG Alexa Fluor 405-conjugated (Abcam, Cambridge, UK), donkey anti-goat IgG-FITC-conjugated and donkey anti-mouse IgG Alexa Fluor 488-conjugated (Abcam) were used as secondary antibodies. If not indicated otherwise, antibodies were purchased from Santa Cruz Biotechnology (Dallas, TX, USA). Data were obtained using a Guava^®^ easyCyte Flow Cytometer 400 (Merck KGaA, Darmstadt, Germany).

### 4.4. Proteome Profiler™ Array

A Proteome Profiler™ Human Phospho-Kinase Array Kit (R&D Systems GmbH, Wiesbaden-Nordenstadt, Germany) was performed to screen MCF-7 and MDA-MB-231 cells for changes in intracellular signaling pathways comparing untreated cells with the effects of COL1 binding and cytotoxic stress by MX (EC_50_). Cell lysates from MCF-7 cells were prepared and Pierce™ BCA Protein Assay Kit (LifeTechnologies, Thermo Fisher Scientific Inc.) was used to quantify total protein. The assay was performed according to the manufactures’ instructions. Membranes were photographed and quantified using ChemiDoc XRS+ imaging acquiring system (BioRad, Hercules, CA, USA), and Image Lab software v. 6.0 (BioRad).

### 4.5. Western Blot

Cells extraction, lysate quantification, SDS-Page and western blots were performed as described using stain-free gels [20]. Membranes were incubated with rabbit anti-β-catenin, mouse anti-GAPDH (GeneTex), goat anti-pGsk-3β (Ser9), rabbit anti-LaminB1, mouse anti-pCREB (Ser133), mouse anti-pMEK1/2, mouse anti-β-actin, rabbit anti-FAK, rat anti-pFAK (Y397, R&D Systems) rabbit anti-PI3K, goat anti-pPI3K (Tyr 508), goat anti-AKT1, rabbit anti-pAKT1 (Tyr308), rabbit anti-ERK1/2 (Cell Signaling Technology, Frankfurt am Main, Germany), rabbit anti pERK1/2 (Thr202/Tyr204, Cell Signaling Technology) as well as goat anti-rabbit, goat anti-rat, donkey anti-goat and anti-mouse IgG kappa binding protein IgG HRP-conjugated diluted in 1% BSA solution. If not indicated otherwise antibodies were purchased from Santa Cruz Biotechnology. Western blots were quantified via chemiluminescence using a Clarity Western ECL substrate chemiluminescence kit (BioRad). Besides the loading control β-Actin, we used stainfree total protein normalization. Membranes were photographed and quantified using ChemiDoc XRS+ imaging acquiring system (BioRad) and Image Lab software v. 6.0 (BioRad).

### 4.6. TOP-Flash Assay

Cells were seeded one day before transfection into a 24-well plate in 0.5 mL cell culture medium at a density of 4 × 10^4^. Thereafter, medium was changed to medium free of additives and transfection reagent and DNA were added at a ratio of 1:1. Cignal TCF/LEF Reporter Assay Kit (LUC) vectors (Quiagen, Hilden, Germany) were used as DNA constructs. The transfection reagent FuGENE^®^ HD (Promega, Mannheim, Germany) was used following the manufacturers’ protocol. Cells were incubated for 24 h, then medium was changed to fresh DMEM containing additives for further 8 h before an incubation was started for another 72 h. Cells were incubated on COL1, with Mn(II) or with the combination of those two before incubation with MX or CDDP, respectively for 72 h. 12 h prior to the luminescence measurement, LiCl (AppliChem GmbH, Darmstadt, Germany) was added at a concentration of 500 mM as positive control for Wnt signaling activity. Treatments were measured in duplicates with *n* = 3.

Luminescence intensity was determined using ONE-Glo™ Luciferase Assay System (Promega) and Renilla-Glo^®^ Luciferase Assay System (Promega) after lysing cells with 100 µL of Glo Lysis Buffer (Promega) and splitting the resulting lysates in two wells of a white 96-well plate. To one portion of lysate, 50 µL of ONE-Glo™ Luciferase Assay System was added and 50 µL of Renilla-Glo^®^ Luciferase Assay System to the other. Both wells were measured according to manufactures’ protocol using a FLUOstar Optima from BMG Labtech. Results from the Renilla luminescence were used for normalization, by selecting an arbitrary value as centre of reference to calculate normalization factors. Resulting normalized firefly luciferase values where analyzed for statistical significance.

### 4.7. β1-Knockdown

β1-Integrin of MCF-7 cells was knocked down by using viral transduction. Cells were seeded at a concentration of approximately 1000 cells per well of a 96-well plate and incubated overnight in DMEM medium containing additives. The next morning, polybrene was added at a concentration of 5 µg/100 µL and 12 µL of β1-integrin or scrambled control shRNA (h) lentiviral particles were added according to manufactures protocol (Santa Cruz Biotechnology) and calculated from the recommended lentivirus MOI (multiplicity of infection) for MCF-7cells. The next day, media was changed and the still infectious medium containing lentiviral particles (Santa Cruz Biotechnology) were used to transfect another well in the same manner. The transfected cells were further incubated in fresh medium containing 0.3 µg/mL puromycin (Carl Roth GmbH) until they nearly reached confluence and then passaged for further experiments. Knock-down was confirmed by Western blot using a mouse anti-β1-integrin antibody (P2D5, Santa Cruz).

### 4.8. Statistical Analysis

Comparisons were performed using the software Prism™ (GraphPad Software, San Diego, CA, USA). MTT results were analyzed by nonlinear regression (four parameters, variable slope) to obtain sigmoidal dose-response curves and to determine the EC_50_ at the curves inflexion point. From the ratio between the EC_50_ of treated cells (resistant) by the EC_50_ of control cells (sensitive) the RF (resistance factor) is calculated. Moreover, statistical analysis was performed using either one way ANOVA and Dunnett’s test or using paired t-test (* *p* < 0.05; ** *p* < 0.01; *** *p* < 0.001).

## 5. Conclusions

Data of this study provide evidence for the role of CAM-DR in breast cancer cells to attenuate the sensitivity to the guideline based cytotoxic drugs MX and CDDP. The elucidated signaling pathways involved in translating a matrix binding into reduced sensitivity display intracellular pathway components as promising targets for sensitization strategies. These are FAK/PI3K/AKT pathway components in MCF-7 cells, MAPK pathway molecules in MDA-MB-231 cells. Loss of β1-integrin leads to a signaling shift from FAK/PI3K/AKT to MAPK in MCF-7 cells. Among both MCF-7 and MDA-MB-231, the pathways’ shared signaling molecule pCREB appears as a highly deregulated and thus promising target.

## Supplementary Materials

The following is available online at http://www.mdpi.com/2072-6694/10/12/495/s1. Figure S1: Western blot analysis of cytosolic β-catenin in MDA-MB-231 cells shows no significant differences upon treatment with COL1 or MX, precluding Wnt pathway, Figure S2: Cytotoxicity studies of used inhibitors by MTT based cell viability assay in MCF-7 and MDA-MB-231 cells, Figure S3: Inhibition of FAK and PI3K and the impact on MDA-MB-231 sensitivity to MX and CDDP cytotoxicity, Figure S4: Cytotoxicity studies of used inhibitors by MTT based cell viability assay in MDA-MB.231 and MCF-7 cells, Figure S5: Pixel densitometry analysis of the Western blots in Figure 7B.
