# Supplementary material for: Mechanisms of Matrix-Induced Chemoresistance of Breast Cancer Cells—Deciphering Novel Potential Targets for a Cell Sensitization
Source: Cancers (Basel). 2018 Dec 6;10(12):495. doi: 10.3390/cancers10120495 (PMC6315379; doi:10.3390/cancers10120495)
Supplement: Supplementary file 1 [file cancers-10-00495-s001.pdf]

Supplementary Materials: Mechanisms of Matrix-Induced Chemoresistance of Breast Cancer Cells—Deciphering Novel Potential Targets for a Cell Sensitization

Bastian Jakubzig, Fabian Baltes, Svenja Henze, Martin Schlesinger and Gerd Bendas

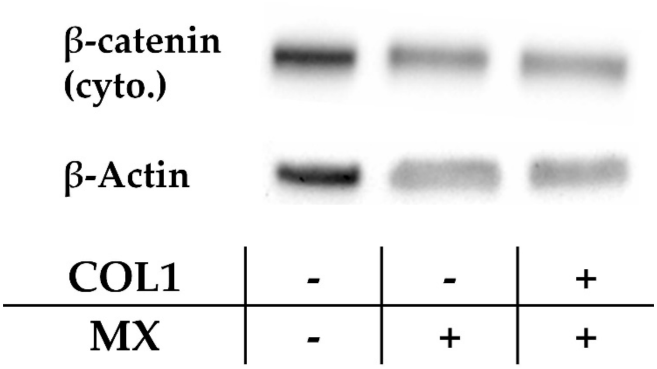

Figure S1. Western blot analysis of cytosolic β-catenin in MDA-MB-231 cells shows no significant differences upon treatment with COL1 or MX, precluding Wnt pathway.

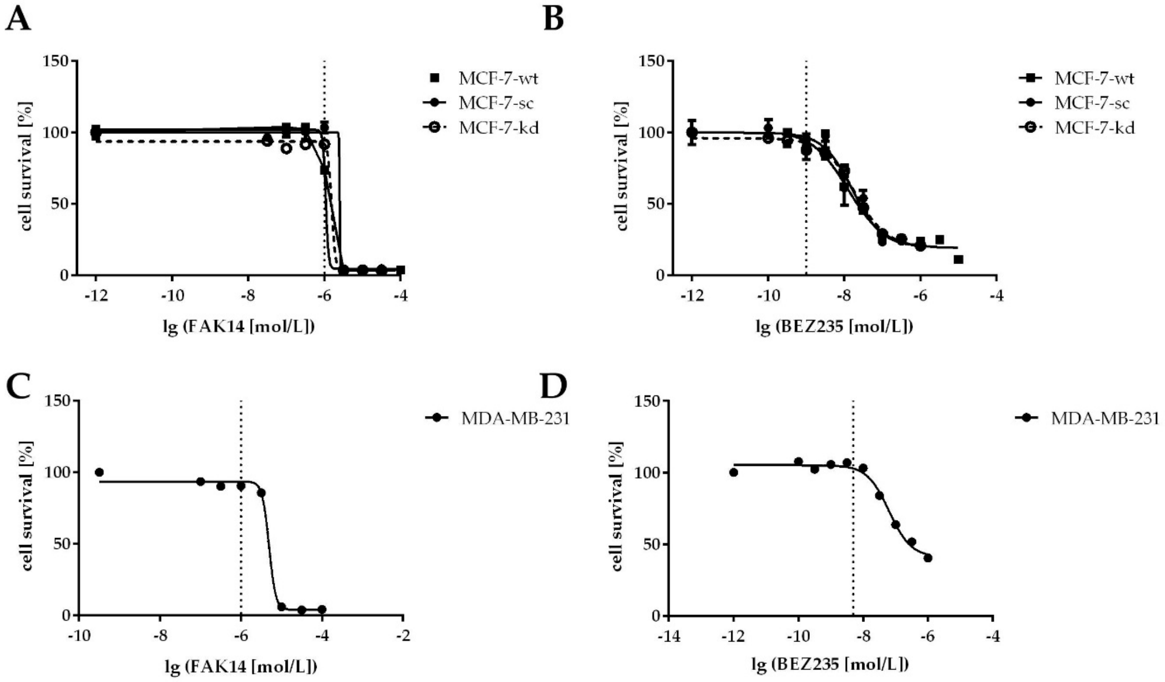

Figure S2. Cytotoxicity studies of used inhibitors by MTT based cell viability assay in MCF-7 (upper part) and MDA-MB-231 cells (lower part). Indicated are the used concentrations. (**A**,**C**) FAK inhibitor FAK14 at 1 µM. (**B**,**D**) PI3K/mTOR inhibitor BEZ235 at 1 nM. All inhibitors displayed no cytotoxicity.

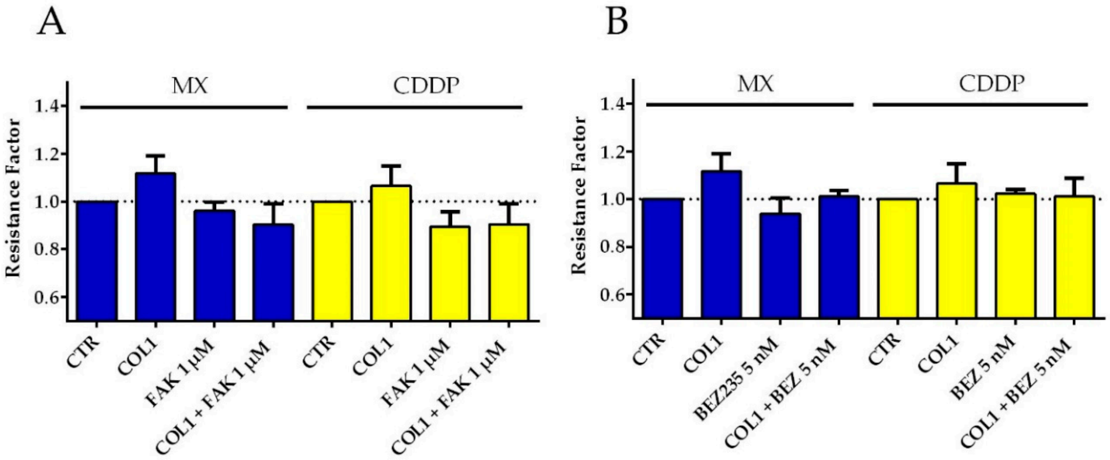

Figure S3. Inhibition of FAK and PI3K and the impact on MDA-MB-231 sensitivity to MX and CDDP cytotoxicity. The RF confirms that (**A**) inhibition of FAK in MCF-7 cells by FAK14 (1 µM) increases sensitivity against MX (blue) and CDDP (yellow). (**B**) Inhibition of PI3K by BEZ235 (5 nM) displays no additional effect to MX and CDDP treatment.

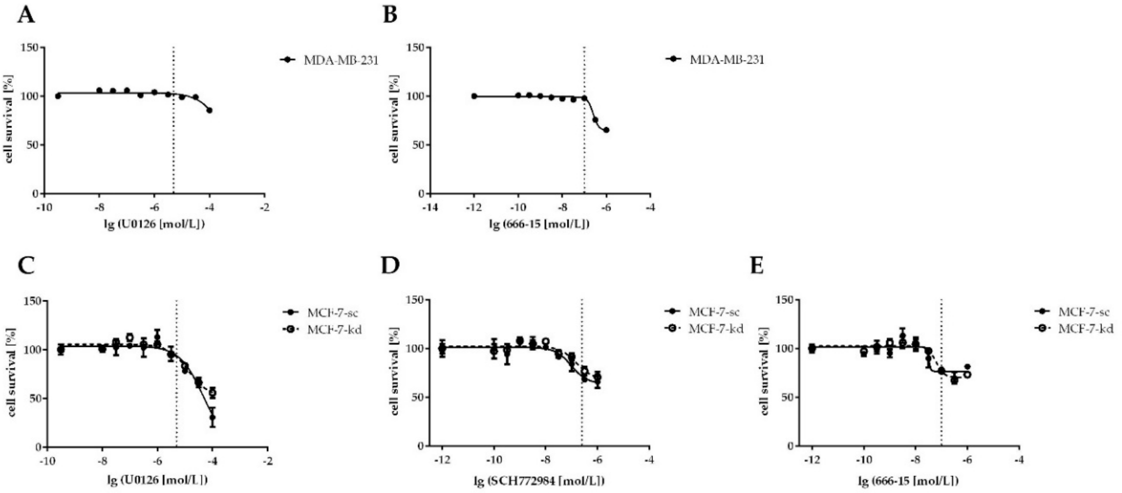

Figure S4. Cytotoxicity studies of used inhibitors by MTT based cell viability assay in MDA-MB.231 (upper part) and MCF-7 cells (lower part). Indicated are the used concentrations. (**A**,**C**) MEK inhibitor U0126 at 5 µM. (**B**,**E**) ERK inhibitor SCH772984 at 250 nM. (**D**) CREB inhibitor 666-15 at 100 nM. All inhibitors displayed no or neglectable cytotoxicity.

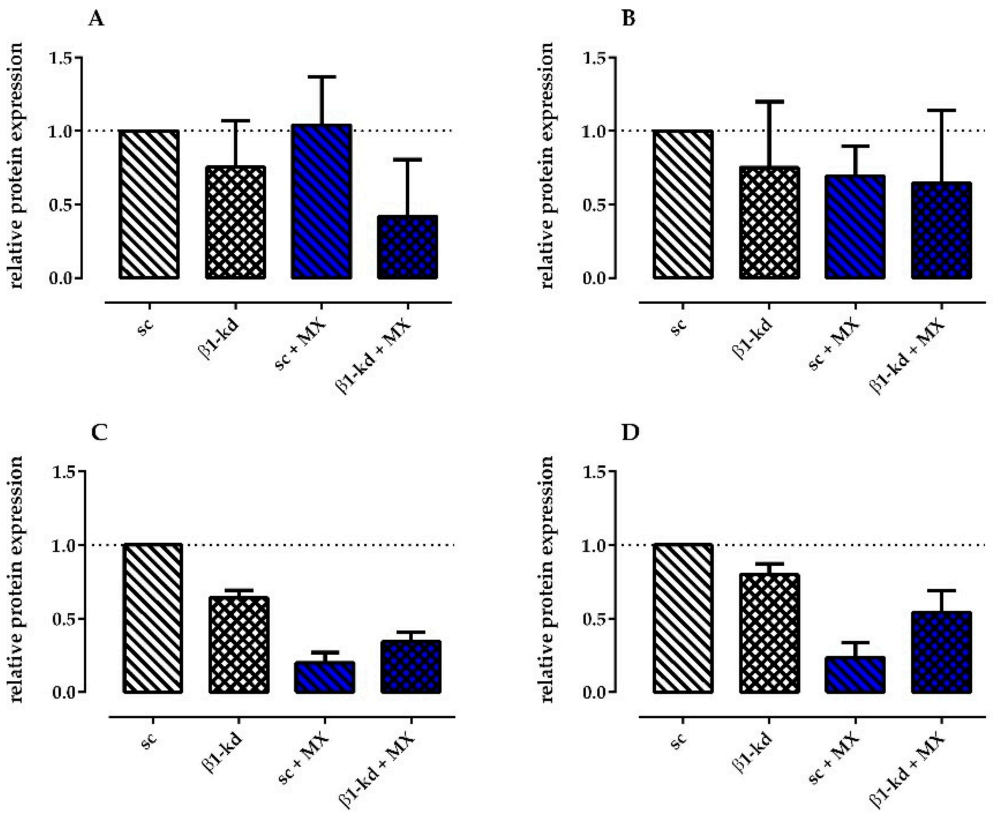

Figure S5. Pixel densitometry analysis of the Western blots in Figure 7B of: (**A**) pCREB (**B**) pMEK1/2 (**C**) pERK 1 and (**D**) pERK 2. Blots were normalized using stainfree technology and all treatments are displayed in relation to the untreated MCF7-sc cells.
